# Neonatal mortality in Kenyan hospitals: a multisite, retrospective, cohort study

**DOI:** 10.1136/bmjgh-2020-004475

**Published:** 2021-05-31

**Authors:** Grace Irimu, Jalemba Aluvaala, Lucas Malla, Sylvia Omoke, Morris Ogero, George Mbevi, Mary Waiyego, Caroline Mwangi, Fred Were, David Gathara, Ambrose Agweyu, Samuel Akech, Mike English, Clinical Information Network authors, Mercy Chepkirui

**Affiliations:** 1 Department of Paediatrics and Child Health, University of Nairobi, Nairobi, Kenya; 2 Health Services Unit, KEMRI – Wellcome Trust Research Institute, Nairobi, Kenya; 3 Health Services, Nairobi Metropolitan Services, Nairobi, Kenya; 4 Division of Neonatal and Child Health, Kenya Ministry of Health, Nairobi, Kenya; 5 Kenya Paediatric Research Consortium (KEPRECON), Nairobi, Kenya; 6 MARCH Centre, London School of Hygiene and Tropical Medicine, London, UK; 7 Nuffield Department of Clinical Medicine, Oxford, Oxfordshire, UK

**Keywords:** epidemiology, health services research, paediatrics, cohort study

## Abstract

**Background:**

Most of the deaths among neonates in low-income and middle-income countries (LMICs) can be prevented through universal access to basic high-quality health services including essential facility-based inpatient care. However, poor routine data undermines data-informed efforts to monitor and promote improvements in the quality of newborn care across hospitals.

**Methods:**

Continuously collected routine patients’ data from structured paper record forms for all admissions to newborn units (NBUs) from 16 purposively selected Kenyan public hospitals that are part of a clinical information network were analysed together with data from all paediatric admissions ages 0–13 years from 14 of these hospitals. Data are used to show the proportion of all admissions and deaths in the neonatal age group and examine morbidity and mortality patterns, stratified by birth weight, and their variation across hospitals.

**Findings:**

During the 354 hospital months study period, 90 222 patients were admitted to the 14 hospitals contributing NBU and general paediatric ward data. 46% of all the admissions were neonates (aged 0–28 days), but they accounted for 66% of the deaths in the age group 0–13 years. 41 657 inborn neonates were admitted in the NBUs across the 16 hospitals during the study period. 4266/41 657 died giving a crude mortality rate of 10.2% (95% CI 9.97% to 10.55%), with 60% of these deaths occurring on the first-day of admission. Intrapartum-related complications was the single most common diagnosis among the neonates with birth weight of 2000 g or more who died. A threefold variation in mortality across hospitals was observed for birth weight categories 1000–1499 g and 1500–1999 g.

**Interpretation:**

The high proportion of neonatal deaths in hospitals may reflect changing patterns of childhood mortality. Majority of newborns died of preventable causes (>95%). Despite availability of high-impact low-cost interventions, hospitals have high and very variable mortality proportions after stratification by birth weight.

**Key questions**

**What is already known?**

- Quality of inpatient care for small and sick newborns in low-income and middle-income countries (LMICs) is poor.
- Poor routine data undermines efforts to monitor and promote improvements in the quality of newborn care across hospitals in LMICs.

**What are new findings?**

- Neonates (aged 0–28 days) account for almost half and two-thirds of the admissions and deaths, respectively, in the age group 0–13 years in Kenyan county hospitals.
- Neonatal fatality rate in newborn units (NBUs) is high and variable across hospitals and birth weight categories. Nevertheless, meaningful comparison of neonatal morbidity and mortality across different hospitals in low-resource settings is a major challenge.
- Lack of technologies to accurately estimate the gestation age (GA) makes birth weight categories preferred over GA when stratifying the risk of neonatal mortality. However, use of birth weight is inaccurate as low birth weight conflates both premature birth and the occurrence of small for gestational age that carry different risks of mortality.
- Inconsistent admission criteria in NBUs and kangaroo mother care (KMC) wards, compounded by limited or lack of KMC facilities, may lead to admission of stable preterm babies to the NBUs thus lowering the neonatal fatality rates.

**What do the new findings imply?**

- To allow for comparison of NBU fatality rates across different settings in addition to developing well-functioning information systems that operate at scale, there is need to harmonise: (1) GA assessment methods, (2) data capture on early neonatal deaths that occur in labour wards, (3) diagnostic criteria for common neonatal conditions and (4) standardise NBU and KMC admission criteria.

## Introduction

Two and half million neonates die annually accounting for 47% of all under-five deaths, with 98% of the neonatal deaths occurring in low-income and middle-income countries (LMICs).^1^ Most of these deaths can be prevented through universal access to basic high-quality health services including essential facility-based inpatient care. To improve population health, Kenya has embraced universal health coverage and increased facility-based deliveries to 86%, although with wide geographic disparities (46%–99%).^2^ However, in many LMICs, poor routine data limit our understanding of neonatal outcomes among those requiring hospital admission. This challenges evidence-based planning and resource allocation and undermines data-informed efforts to monitor and promote improvements in the quality of newborn care across hospitals.^3^ Such data are important in Kenya where hospital management has been devolved to county (subregional) governments that vary in their fund allocations to maternal, newborn and reproductive health.^4^ Ideally, individual patient-level data would enable analysis of service use and outcomes and indicate which conditions or facilities should be the targets of improvement interventions, including allocation of human resources. Exploring variation in mortality might also help us understand specific regional risks or learn improvement lessons from positive deviants.^5^ To begin to address some of these information needs, we established the Clinical Information Network for neonates (CIN-Neonatal) building on a successful model established for paediatric wards^6^ and early work in one urban newborn unit (NBU).^3 7 8^ We use routine CIN-Neonatal data from a 2-year period spanning 16 Kenyan county hospitals to describe current neonatal mortality and morbidity patterns in non-tertiary settings.

## Methods

### Study design and participants

We conducted a retrospective cohort study that used paediatric and neonatal inpatient data between 1 April 2018 and 31 March 2020. CIN-Neonatal data spans 16 purposively selected public hospitals (H1–H16) in 12 of the 47 counties in Kenya. For the purposes of some analyses, we also use inpatient records of all children aged 0–13 years including those admitted to hospitals’ general paediatric wards in 14 hospitals (H1–H14). We excluded data from H15 and H16 in the analysis of the burden of all neonatal admissions and mortality among all admissions 0–13 years because only NBU data were collected from these two hospitals. Kenya introduced a curfew and other measures to control the SARS-CoV-2 pandemic on 27 March 2020 and had 59 confirmed cases among all age groups by 31 March 2020. Our data are unlikely to have been affected by the pandemic.^9^

### Setting

#### CIN-Neonatal

The Clinical Information Network (CIN) was established in 2013 as a collaboration between the Ministry of Health (MoH), Kenya Paediatric Association, KEMRI Wellcome Trust Research Programme, University of Nairobi and participating hospitals. The aim of CIN is to improve quality of patients’ data and their utilisation; it had an initial focus on general paediatric wards.^10 11^ CIN expanded stepwise in 2018 to hospitals’ NBUs,^8^ which led to two functionally and administratively linked networks: CIN-Paediatrics (CIN-Paeds) and CIN-Neonatal. CIN-Paeds generates data from the general paediatric wards (all medical admissions of children 0–13 years in hospitals H1–H14), while CIN-Neonatal generates data from 16 hospitals’ NBUs’ admissions. In each hospital, three staff act as focal persons to link the researchers and hospital teams: the paediatrician, the nurse in charge of the NBU and the senior health records information officer. They promote sustained use of codesigned structured patient record forms and encourage better documentation of clinical practices that enable high-quality retrospective data collection.^6 10 11^ The county hospitals are administratively and financially supported by their respective county governments, including their complement of health workers, supply of drugs and equipment.

#### Development of data collection tools and study procedures

In 2014, we began development of neonatal data collection tools and procedures in a large hospital providing exclusive maternity and neonatal services.^8^ We further piloted the data collection tools and methods in three county hospitals from June 2017 revising the tools iteratively to obtain those deployed in 2018. These include structured newborn admission record (NAR) and NBU exit forms that are endorsed by MoH and that are primary data sources for this study. CIN-Neonatal uses data collection and quality assurance methods that are fully described elsewhere.^6 10^ In brief, CIN-Neonatal supports one data clerk in each hospital to abstract biodata, admission and discharge diagnoses, and outcome (alive or dead) from the paper hospital records each day for all patients after discharge. The data are entered directly into a non-proprietary Research Electronic Data Capture tool with inbuilt range and validity checks.^12^ Data entry is guided by a standard operating procedure manual and error-checking systems that form the basis of the data clerks’ training. To ensure no record is missed, the research team benchmarks the admission numbers entered in the CIN-Neonatal database with the aggregate statistics submitted to MoH. External data quality assurance is done by research assistants who visit each hospital every 3 months and re-enter data from 5% of randomly selected records to check consistency with the data clerks’ entries. The overall concordance of the external data quality audits has been ranging between 87% and 92%. Feedback is given to the data clerks and any challenges addressed for continuous improvement of data quality.

We obtained basic descriptive data on hospital infrastructure and resources from the Kenya Harmonized Health Facility Assessment conducted in 2018/2019, the District Health Information System version 2 and directly from CIN’s focal persons.^13^

#### Expansion of the CIN-Neonatal

By April 2018, 12 hospitals had joined CIN-Neonatal. One hospital (H7), though in CIN-Paeds, was not enrolled until November 2018, and with guidance from MoH, one other hospital was added in November 2018 (H15) and two in December 2018 (H1 and H4). These three new hospitals were selected on the basis of having relatively high-volume NBUs. All the 16 NBUs aim to provide specialty care for small and sick newborns with capacity to provide bag and mask ventilation and oxygen, use of radiant warmers or incubators (often shared), intravenous antibiotics, intravenous fluids and nasogastric or orogastric breast milk feeding, kangaroo mother care (KMC) and phototherapy. All the 16 NBUs are higher volume non-tertiary facilities that are aspiring to reach the intermediate level of neonatal care.^14 15^

### Study analysis procedures

We use CIN-Neonatal and CIN-Paeds’ data to create two populations, population A and population B, for analyses.

**Figure 1:**
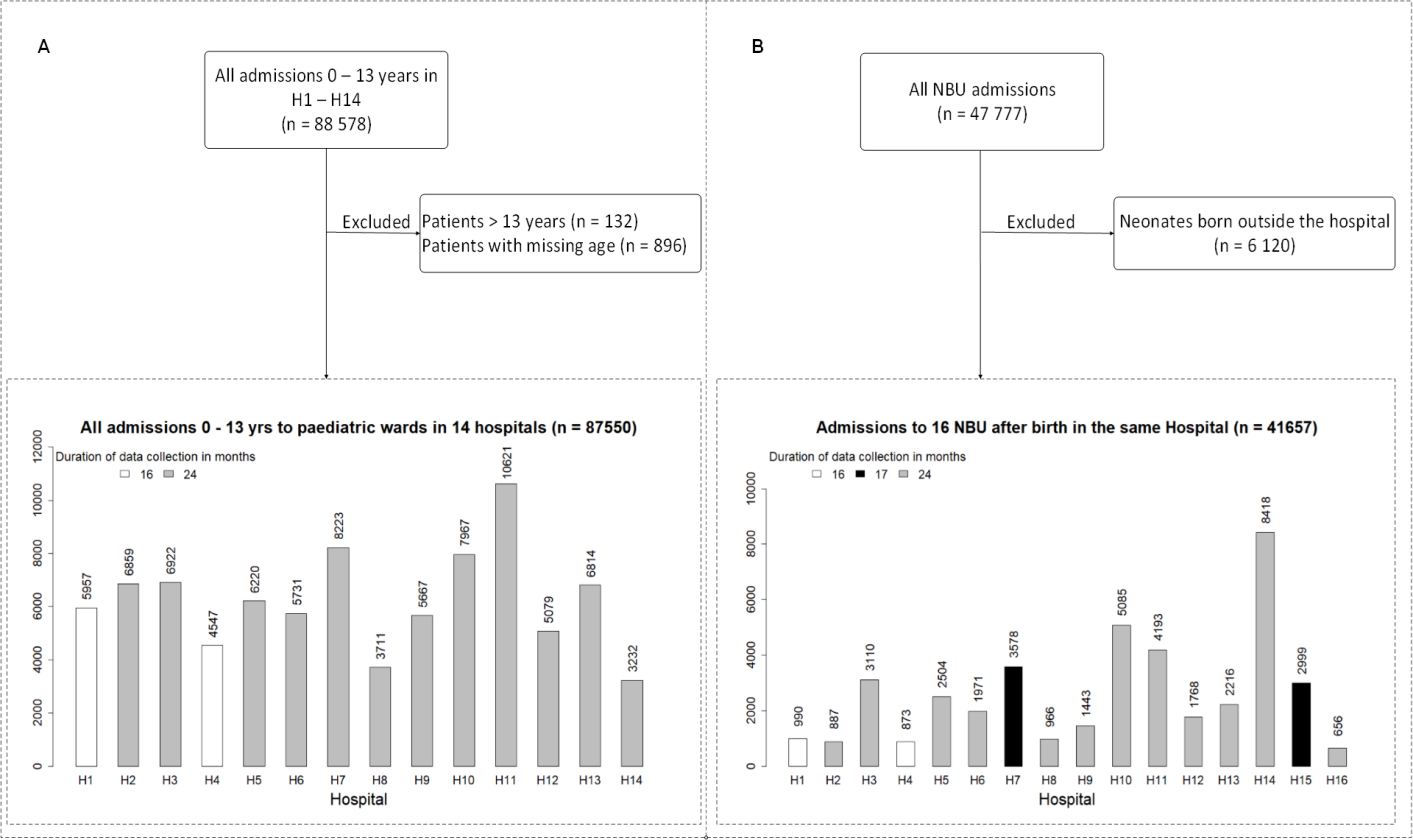
(A) Entire patient population aged 0–13 years admitted in H1–H14 during study period (population A). (B) Inborn newborns admitted in the 16 NBUs during the entire study period (Population B). NBU, newborn unit.

Population A: includes hospital admissions that contribute to both the CIN-Neonatal and CIN-Paeds databases (H1–14), thus excluding data for H15 and H16. We use data for all ages (0–13 years) to show the proportion of all admissions and deaths in county hospitals that are in the neonatal age group (figure 1A).

Population B: includes only neonates admitted to all the 16 NBUs who are also born in the same hospital, thus excluding outborn neonates. We defined an ‘outborn’ neonate as one either born at home, referred into the NBUs from another facility or admitted from home after discharge from a facility-based birth. We excluded all outborn neonates from this analysis because our aim was to examine a relatively comparable population of newborns from each hospital. We used this population to describe characteristics of all inborn neonates disaggregated by hospital and examine morbidity patterns, mortality rates stratified by birth weight and inpatient survival over time (figure 1B).

For analysis of the inborn neonates, population B, we used the birthweight categories described by the WHO^16^ but expanded this to include those 1500–1999 g and 2000–2499 g as separate categories as our earlier work showed these groups have considerably different outcomes.^7^ We used funnel plots to illustrate variation in mortality by birthweight category across hospitals. We plotted cumulative probability of mortality using a competing risks approach to examine in-hospital mortality for inpatient stays of up to 4 weeks postnatal age stratified by birth weight. Data were not available on outcomes after a baby’s discharge if this occurred before 28 days of age. We made several adaptations to our analysis approach due to limited diagnostic capacity in LMICs (box 1).

Box 1 **Adaptation in analysis approach due to limited diagnostic capacity in low income setting**

1. We preferred to use birth weight over gestation age (GA) to stratify our analysis because GA data are often missing and when recorded suffers from the following limitations: (1) it is often estimated from mothers’ report of last menstrual period, (2) routine first trimester ultrasound estimation is rarely done in Kenyan public hospitals and (3) use of recognised clinical assessment tools for estimating gestation are not in regular use.^7^
2. Morbidity patterns are based on all causes of illness/reasons for admission on NBU as documented in the neonates’ admission and/or discharge notes by clinicians. All the NBUs have structured admission records and discharge summary forms that promote the use of common terms for common diagnoses/illness classification. We therefore neither attempted to refine the diagnoses made by the attending clinicians nor to assign a single cause of admission or death. A large majority of diagnoses are based on clinical features supported by only infrequent access to a limited range of diagnostic tests. Some diagnoses imply clinical syndromes or simply reason for admission to NBU rather than formal diagnoses based on the International Classification of Diseases (ICD)-10 system. Thus, our morbidity/reason for admission analysis allows for more than one diagnosis per neonate and thus primarily indicates the need for services or interventions.
3. In the specific case of a recorded diagnosis of low birth weight or preterm birth or of being HIV exposed, we considered these as the admission diagnosis only if no comorbidity was recorded. We describe the former as ‘uncomplicated low birth weight or preterm birth’ if no comorbidity.
4. Clinical diagnoses of birth asphyxia, hypoxic ischaemic encephalopathy and meconium aspiration were collectively categorised as ‘intrapartum related complications’.
5. The day of birth was considered as day 1. There was poor documentation of time of admission in the NBU.

### Patient and public involvement

This study uses routine secondary data. Although members of the public/patients provided routine data required for care provision, no patients or the public were directly involved in the design, conduct, reporting or dissemination plans of this research.

## Results

Characteristics and resources for newborn care in the 16 CIN hospitals are described in table 1. Data on 54 668 neonates were available from the hospitals: 12 hospitals contributed data from 1 April 2018, two hospitals from November 2018 (H7 and H15) and two from December 2018 (H1 and H4) until 31 March 2020, a total of 354 hospital-months.

**Table 1:** Human resource, equipment and supplies in 16 NBUs in the CIN hospitals (H1–H16)

	H1	H2	H3	H4	H5	H6	H7	H8	H9	H10	H11	H12	H13	H14	H15	H16
Deliveries per year*	6387	4441	6228	4581	5515	2945	9939	2578	6744	8641	11 404	5571	5131	3653	8872	21 608
Number of still births (%)*	180 (3)	195 (4)	172 (3)	150 (3)	203 (4)	42 (1)	213 (2)	47 (2)	191 (3)	231 (3)	237 (2)	169 (3)	87 (2)	105 (3)	196 (2)	521 (2)
Number of all NBU admissions per year†	1250	665	1765	902	1516	1047	2640	416	996	2661	2386	868	1394	420	2962	4769
% of outborn neonates among all NBU admissions per year†	36	17	6	25	24	14	3	0	17	1	3	1	8	7	21	7
Number of MOs dedicated to NBU‡	0.5	0.5	0,5	1	1.5	0.5	0.5	0.5	0.5	1	0	0.5	1	0.5	0	5
Number of Paediatricians dedicated to NBU‡	0.5	0.5	1	0.5	1	0.5	1	0.5	0.5	1	1	1	0.5	0.5	1	6
Nurse per day shift§	2	1	1	5	6	2	3	3	3	5	4	2	3	2	3	5
Nurse per night shift§	1	1	2	2	3	2	2	1	1	3	3	1	2	1	2	3
Cots in NBU	17	2	41	23	40	17	39	1	10	53	15	4	32	0	60	50
Babies share cots	yes	yes	no	no	yes	yes	yes	no	yes	no	yes	yes	no	no	no	yes
Incubators¶	10	2	8	10	7	6	8	3	4	7	8	11	6	6	11	7
Babies share incubators	yes	yes	yes	no	yes	yes	yes	yes	Yes	no	yes	yes	no	yes	yes	yes
KMC beds**	10	8	0	7	8	5	0	0	7	4	6	4	2	5	4	16
CPAP machine¶	2	1	0	1	1	0	1	1	0	1	4	4	3	1	4	2
Pulse oximeter¶	2	1	1	1	1	0	2	1	1	0	4	1	3	4	4	4
Ability to do cultures††	limited	no	no	limited	no	limited	limited	no	limited	limited	no	no	limited	no	no	no
Birth weight (g) below which stable LBW are admitted in NBU	2100	2000	2000	2000	2000	2000	2000	1800	1800	2000	2000	1800	1800	1800	2000	1800
Weight required to initiate KMC for stable babies (g)	1500	1200	N/A	1500	1700	1200	N/A	N/A	1300	1200	1200	1500	N/A	1500	1200	1400
Discharge weight from KMC unit (g)	2000	2000	N/A	1800	1900	2000	N/A	N/A	1800	1800	1800	1800	N/A	1800	1800′	1800

Deliveries and NBU admissions January 2019–December 2019.

*Deliveries and still births per year (percentage still births): January 2019–December 2019. Source: District Health Information System 2.

†All NBU admissions (inborn and outborn neonates) and % of outborn neonates in NBU per year: January 2019–December 2019. Source: CIN-Neonatal Database.

‡MO/paediatricians dedicated to NBU: fraction time spent in NBU, 0.5 of person implies that the staff work 50% time of 08:00–17:00 working days in the NBU. 50% of the working period: the staff is in the other paediatric wards.

§Nurses: includes neonatal nurses (NNs) in seven hospitals (H4, H14 and H15 had one NN each, H7 and H11 had two NNs and H16 had three NNs).

¶Functional equipment as per March 2020.

**KMC bed: any type of bed used, including ordinary ward bed, for continuous KMC.

††Ability to do culture: all hospitals able to do diagnostic cultures had erratic supplies of reagents, unreliable culture results and blood culture specimens were not accepted in the laboratories at night.

CPAP, continuous positive airway pressure; KMC, kangaroo mother care; LBW, low birth weight; LOA, length of admission; MO, medical officer (excluded MO interns); N/A, not applicable (continuous KMC not practised); NBU, newborn unit.

### Burden of all neonatal (population A; inborn and outborn) admissions and mortality among all admissions 0–13 years in 14 hospitals (H1–H14)

**Figure 2:**
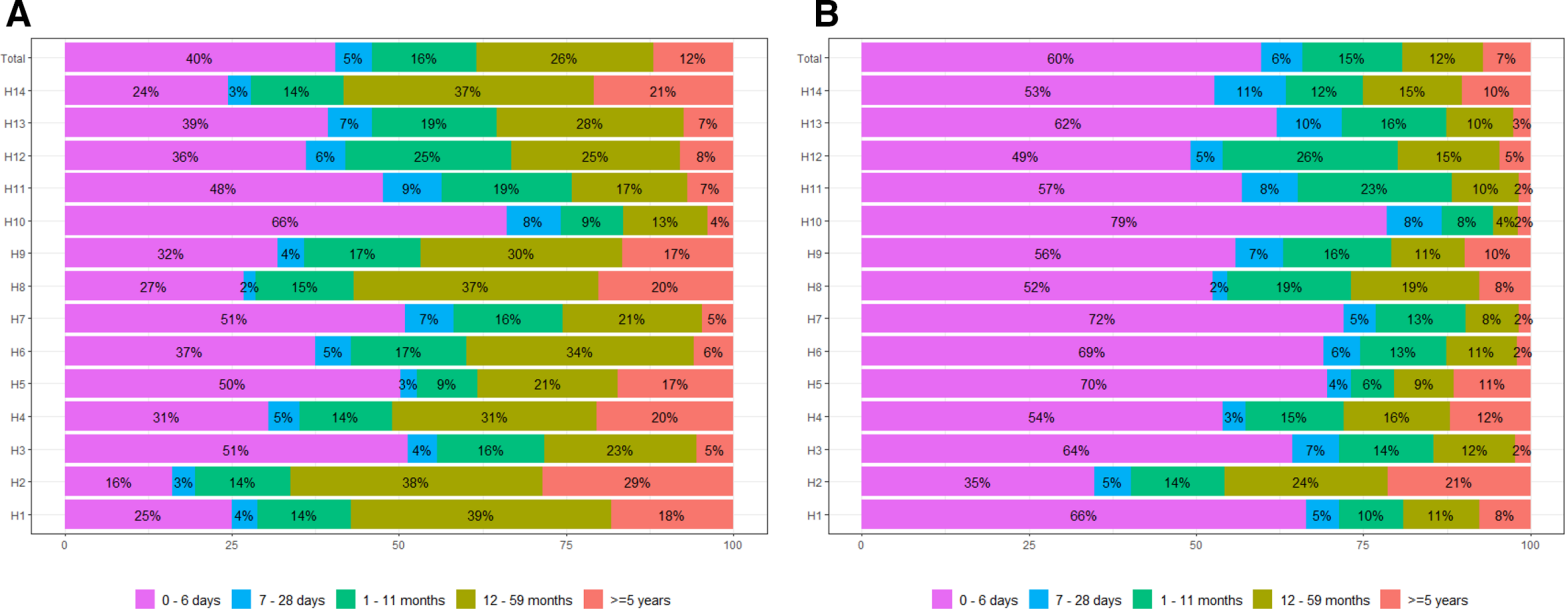
(A) Admissions in the 14 CIN hospitals of all patients aged 0–13 years (population A) disaggregated in age categories. (B) Mortalities in the 14 CIN hospitals of all patients aged 0–13 years (population A) disaggregated in age categories. CIN, Clinical Information Network.

During the study period, a total of 88 578 patients were admitted in the NBUs and general paediatrics wards of the 14 hospitals (excluding H15 and H16) that contribute to both CIN-Neonatal and CIN-Paed data. After excluding 896 patients whose age was missing and 132 who were aged >13 years, we obtained 87 550 patients aged 0–13 years (population A) (figure 1A). Among these patients, 40 183 (46%) were aged 0–28 days, with 88% (35 416/40 183) of the neonates aged 0–6 days. The percentage of neonatal admissions among the inpatient population aged 0–13 years across the 14 hospitals widely varied (range 20%–74%) (figure 2A).

Overall, 9% (7806/87 550) of all patient admissions aged 0–13 years in these hospitals died. Neonates (0–28 days old) comprised of 66% (5142/7799) of these deaths, with 91% (4668/5142) of the neonatal deaths occurring among neonates aged 0–6 days. The contribution of neonatal deaths to all inpatient deaths aged <13 years also varied widely across hospitals (range 40%–87%) (figure 2B).

### Characteristics of the inborn newborns admitted to NBUs of 16 hospitals (population B)

After excluding 6120 outborn neonates, we obtained data on 41 657 inborn neonates (aged 0–28 days) admitted in NBUs across the 16 hospitals during the study period (figure 1B). In table 2, we describe characteristics of the 41 657 neonates (population B). The denominator for each variable excludes neonates with missing information for the corresponding variable. Females accounted for 45% (18 411/41 112) of all NBU admissions (range 42%–48% across the hospitals). Overall, 90% (36 603/40 851) of inborn NBU cases were admitted on their first day of life. Gestational age (GA) was recorded by the admitting clinician in 84% (35 009/41 647) cases. Based on GA estimates, 30% (10 666/35 009) of these neonates were preterm (gestation age <37 weeks), with 3% (926/35 009), 7% (2521/35 009), 6% (2162/35 009) and 14% (5057/35 009) of admissions with GA <28 weeks, 28–<32 weeks, 32–<34 weeks and 34–<37 weeks, respectively. Likewise, 30% (12 202/41 166) of NBU admissions who had birth weight documented were low birth weight (<2500 g). Out of the 41 166 neonates with birth weight documented, 2% (range across hospitals 1%–4%) had birth weight <1000 g, and 5% (4%–11%), 11% (7%–18%) and 12% (9%–14%) had birth weight 1000–1499 g, 1500–1999 g and 2000–2499 g, respectively.

**Table 2:** Characteristics of NBU admissions of all inborn neonates (population B) admitted in 16 NBUs in the CIN during the study period

Total admissions	Total	H1	H2	H3	H4	H5	H6	H7	H8	H9	H10	H11	H12	H13	H14	H15	H16
	41 657	990	887	3110	873	2504	1971	3578	966	1443	5085	4193	1768	2216	2999	656	8418
Gestation period documented	n=35 009	n=788	n=725	n=2713	n=775	n=2178	n=1357	n=3382	n=823	n=1304	n=4473	n=3595	n=1343	n=2031	n=2182	n=517	n=6823
<28 weeks (%)	926 (3)	29 (4)	31 (4)	39 (1)	41 (5)	102 (5)	24 (2)	56 (2)	33 (4)	43 (3)	85 (2)	169 (5)	52 (4)	36 (2)	48 (2)	23 (4)	115 (2)
28–<32 weeks (%)	2521 (7)	82 (10)	91 (13)	109 (4)	80 (10)	261 (12)	102 (8)	200 (6)	78 (9)	131 (10)	237 (5)	362 (10)	157 (12)	101 (5)	171 (8)	56 (11)	303 (4)
32–<34 weeks (%)	2162 (6)	76 (10)	67 (9)	106 (4)	68 (9)	213 (10)	62 (5)	190 (6)	58 (7)	98 (8)	236 (5)	253 (7)	180 (13)	97 (5)	145 (7)	51 (10)	262 (4)
34–<37 weeks (%)	5057 (14)	135 (17)	90 (12)	293 (11)	155 (20)	350 (16)	208 (15)	447 (13)	151 (18)	191 (15)	709 (16)	474 (13)	286 (21)	262 (13)	321 (15)	106 (21)	879 (13)
37–<42 weeks (%)	23 031 (66)	418 (53)	413 (57)	2082 (77)	381 (49)	1157 (53)	933 (69)	2376 (70)	444 (54)	824 (63)	2900 (65)	2208 (61)	627 (47)	1414 (70)	1404 (64)	257 (50)	5193 (76)
≥42 weeks (%)	1312 (4)	48 (6)	33 (5)	84 (3)	50 (6)	95 (4)	28 (2)	113 (3)	59 (7)	17 (1)	306 (7)	129 (4)	41 (3)	121 (6)	93 (4)	24 (5)	71 (1)
Birth weight documented	n=41 166	n=961	n=807	n=3109	n=837	n=2471	n=1941	n=3568	n=955	n=1391	n=5073	n=4170	n=1747	n=2180	n=2933	n=641	n=8382
<1 kg (%)	723 (2)	13 (1)	17 (2)	41 (1)	31 (4)	52 (2)	38 (2)	62 (2)	21 (2)	39 (3)	77 (2)	98 (2)	66 (4)	35 (2)	36 (1)	7 (1)	90 (1)
1–<1.5 kg (%)	2113 (5)	43 (4)	87 (11)	108 (3)	80 (10)	187 (8)	78 (4)	168 (5)	50 (5)	130 (9)	181 (4)	293 (7)	174 (10)	94 (4)	103 (4)	40 (6)	297 (4)
1.5–<2 kg (%)	4362 (11)	142 (15)	123 (15)	204 (7)	132 (16)	337 (14)	178 (9)	335 (9)	108 (11)	236 (17)	390 (8)	566 (14)	306 (18)	206 (9)	260 (9)	109 (17)	730 (9)
2 -<2.5 Kg (%)	5004 (12)	138 (14)	75 (9)	306 (10)	119 (14)	323 (13)	249 (13)	442 (12)	131 (14)	162 (12)	551 (11)	512 (12)	241 (14)	275 (13)	389 (13)	84 (13)	1007 (12)
2.5–4 kg (%)	26 341 (64)	568 (59)	468 (58)	2340 (75)	440 (53)	1275 (52)	1312 (68)	2329 (65)	593 (62)	777 (56)	3616 (71)	2240 (54)	905 (52)	1351 (62)	1849 (63)	380 (59)	5898 (70)
>4 kg (%)	2623 (6)	57 (6)	37 (5)	110 (4)	35 (4)	297 (12)	86 (4)	232 (7)	52 (5)	47 (3)	258 (5)	461 (11)	55 (3)	219 (10)	296 (10)	21 (3)	360 (4)
Mode of delivery documented	n=40 914	n=944	n=823	n=3107	n=856	n=2471	n=1854	n=3569	n=959	n=1433	n=5062	n=4155	n=1748	n=2194	n=2944	n=640	n=8155
Caesarean section (%)	15 726 (38)	289 (31)	216 (26)	1244 (40)	352 (41)	891 (36)	647 (35)	1480 (41)	365 (38)	351 (24)	2409 (48)	1367 (33)	631 (36)	821 (37)	1214 (41)	232 (36)	3217 (39)
Sex documented	n=41 112	n=921	n=887	n=3106	n=846	n=2321	n=1937	n=3559	n=879	n=1440	n=5079	n=4188	n=1763	n=2193	n=2992	n=611	n=8390
Female (%)	18 411 (45)	417 (45)	405 (46)	1408 (45)	406 (48)	1086 (47)	883 (46)	1670 (47)	409 (47)	626 (43)	2236 (44)	1798 (43)	803 (46)	994 (45)	1393 (47)	257 (42)	3620 (43)
HIV exposure documented	n=38 835	n=683	n=756	n=2985	n=746	n=2254	n=1832	n=3464	n=923	n=1324	n=4692	n=4101	n=1643	n=2092	n=2581	n=594	n=8165
HIV positive (%)	1688 (4)	14 (2)	42 (6)	105 (4)	108 (14)	76 (3)	27 (1)	155 (4)	143 (15)	38 (3)	195 (4)	118 (3)	104 (6)	62 (3)	83 (3)	20 (3)	398 (5)
Age documented	n=40 851	n=964	n=803	n=3106	n=865	n=2476	n=1946	n=3564	n=960	n=1429	n=4812	n=4181	n=1699	n=2182	n=2885	n=632	n=8347
≤1 day (%)	36 603 (90)	778 (81)	583 (73)	2956 (95)	804 (93)	2337 (94)	1571 (81)	3383 (95)	866 (90)	1131 (79)	4564 (95)	4154 (99)	1493 (88)	2009 (92)	2500 (87)	575 (91)	6899 (83)
≥2 days (%)	4248 (10)	186 (19)	220 (27)	150 (5)	61 (7)	139 (6)	375 (19)	181 (5)	94 (10)	298 (21)	248 (5)	27 (1)	206 (12)	173 (8)	385 (13)	57 (9)	1448 (17)

CIN, Clinical Information Network; NBUs, newborn units.

Four per cent (1688/38 835) of the neonates in whom HIV maternal status was documented were HIV exposed. Although, in two sites (H4 and H8), about 15% of NBU admissions were HIV exposed.

#### Morbidity patterns among inborn NBU admissions in the 16 hospitals in CIN-Neonatal (population B)

There were 53 047 diagnoses/reasons of NBU admissions recorded at admission and/or discharge among the 41 657 inborn neonates. Five conditions accounted for 80% of the diagnoses in the NBUs. These included intrapartum-related complications (30%), respiratory distress syndrome (18%), neonatal sepsis (15%), jaundice (12%) and uncomplicated low birth weight (LBW)/prematurity (5%). Intrapartum-related complications was the most common (34%, 12 534/37 321) diagnosis among neonates with birth weight ≥2500 g, while respiratory distress syndrome (RDS) was the most common disorder (37%, 3285/8772) among those <2000 g. Neonatal sepsis and jaundice accounted for 17% (6 301/37 312) and 13% (5 009/37 312) of the disease episodes in neonates with birth weight ≥2500 g, respectively, vis-a-vis 9% (832/8772) and 7% (589/8772) of the disease episodes among neonates with birth weight <2000 g (online supplemental figure 1; online supplemental table S1). Comorbidities were common (online supplemental figure 2).

10.1136/bmjgh-2020-004475.supp1 Supplementary data

10.1136/bmjgh-2020-004475.supp2 Supplementary data

10.1136/bmjgh-2020-004475.supp3 Supplementary data

#### Mortality among inborn NBU admissions in 16 hospitals in CIN-Neonatal (population B)

**Figure 3:**
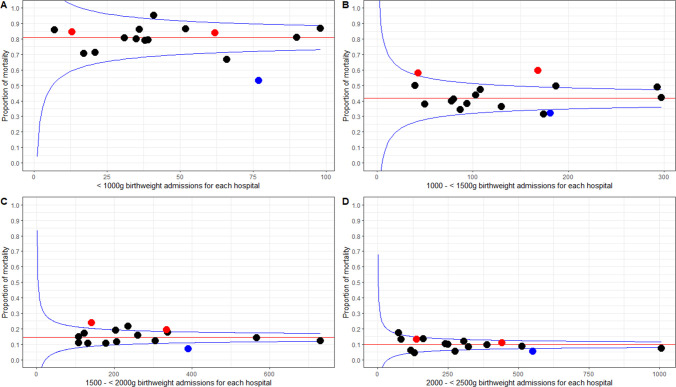
Funnel plots of mortality of inborn newborns by number of inborn newborns admitted in each of the 16 CIN NBUs (population B) during the study period. Plots A, B, C and D depict plots for admissions birth weight category <1000 g, 1000–<1500 g, 1500–<2000 g and 2000–<2500 g, respectively. Each dot represents neonatal mortality of each of the 16 NBUs. Blue dots represent H10 that had consistently lower mortality in all weight categories, and red dots represent H1 and H7 that had consistently higher mortality for babies in the 1000–2000 g range. The red line within the funnel represents the median mortality in the 16 NBUs.

There were 4266 neonates who died among 41 657 inborn neonates admitted in NBUs giving a crude fatality rate of 10.2% (95% CI 9.97% to 10.55%) with wide variation in the hospital-specific neonatal fatality rates (range 5%–14%). Of the babies who died, 4132 (97%) had birth weight documented. In those with a birth weight <1000 g, median mortality was 79% (range 53%–95%), with a marked decrease to 43% (range 32%–60%) for those with birth weight 1000–1499 g and further decreases for those with birth weight 1500–1999 g, 2000–2499 g, 2500–3999 g and ≥4000 g where median mortality rates were 14% (range 7%–24%), 9% (range 5%–17%), 6% (range 3%–12%) and 2% (range 0%–14%), respectively. The median and hospital-specific mortality rates are illustrated using funnel plots. One hospital (H10) and two hospitals (H1 and H7) had consistently lower and higher mortality, respectively, for babies with birth weight 1000–1999 g. The funnel plots suggest a threefold variation in mortality observed for inborn populations with birth weights of 1000–1499 g and 1500–1999 g (figure 3; online supplemental table S2). This may be greater than expected by chance, although with only 16 hospitals contributing to the observations, caution is required in this interpretation. Among the 4132 deaths whose birth weight was documented, 62% (2544/4132) were LBW (<2500 g). Considering the limited capacity to manage extreme LBW (ELBW) and very LBW (VLBW) as shown in table 1, our data raise concern that 64% (2653/4132) of all deaths among the inborns neonates occurred among neonates with birth weight >1500 g, while 22% (911/4132) and 14% (568/4132) were in those with birth weight 1000–1499 and <1000 g, respectively. This may be expected given that 93% (38 330/41 166) of all inborn neonates had birth weight >1500 g.

10.1136/bmjgh-2020-004475.supp4 Supplementary data

Many deaths were among inborn neonates with multiple diagnoses and over 95% were associated with five diagnoses: intrapartum-related complications, uncomplicated prematurity/LBW, RDS, neonatal sepsis and jaundice. As with the pattern observed for morbidity, however, babies with higher birth weights who died were more likely to have a diagnosis of intrapartum-related complications (57% of the 1588 babies with birth weight ≥2500 g who died had a single diagnosis of intrapartum-related complications). In contrast, of the 2107 inborns neonates with birth weights <2000 g who died, 29% had diagnoses of uncomplicated prematurity/LBW, while 35% had prematurity/LBW complicated by RDS (online supplemental figure 2). Data were available for length of stay in NBUs for 98% (4197/4266) of the inborn neonates who died. Of these 4197 deaths, 60% occurred on the first day of admission, 82% and 91% in 0–3 days and 0–6 days, respectively, of admission to NBUs. The probability of death was highest in the first 3 days of admission in all birth weight categories (online supplemental figure 3).

10.1136/bmjgh-2020-004475.supp5 Supplementary data

## Discussion

We sought to describe the characteristics, disease patterns and outcomes of inborn neonates admitted in NBUs in a set of Kenyan counties’ referral hospitals. This study has demonstrated that even in low-resource settings, it is possible to have clinical networks enabling multisite data collection that yields quality data that could inform policy and help in planning newborn services that are context sensitive. Neonates contribute to almost half (46%) of all admissions among medical patients aged 0–13 years, although this proportion considerably varies across hospitals. The reasons for this variation are likely to be complex and depend on admission policies in NBUs and paediatric wards, different disease patterns in paediatrics wards (eg, prevalence of malaria) and local referral patterns especially for outborn neonates.^10^ For example, we have previously demonstrated considerable variation in classification of paediatric illnesses’ severity (eg, malaria and pneumonia) in Kenyan hospitals. This may lead to higher admission rates in paediatric wards in some settings that result in a relatively lower proportion of newborn admissions.^17–19^ Importantly, neonates contribute to two-thirds of the mortality among all patients aged 0–13 years admitted in CIN hospitals. This high proportion of deaths may reflect changing patterns of mortality at a population level in Kenya especially among children aged 1–4 years.^1^ This is important as hospitals in general may not be well equipped, staffed or organised to cater for the needs of the large proportion of small and sick neonates admitted in either paediatric wards or NBUs.^20–22^

Five conditions, all to some degree preventable, accounted for 80% of the disease episodes at admission among inborn neonates in NBUs, with intrapartum-related complications being the most common (30%) cause of admission. Reducing intrapartum-related complications means having a functional health system to provide quality care during the antenatal period, labour, childbirth and immediate postnatal period.^23–25^ Other common causes of admission are LBW/prematurity-related complications such as RDS, a condition that hospitals were poorly prepared to manage. Majority (10/16) of the NBUs had none or just one continuous positive airway pressure (CPAP) machine. Managing RDS with conventional oxygen therapy, as high-income countries (HICs) practised in 1960s, is estimated to be associated with a survival rate of less than half that associated with high-quality care supported by CPAP.^26^

However, while better technologies may help in the management of these common neonatal conditions, they need to be carefully introduced. Where human resources for health (HRH) are limited, adding new tasks can exacerbate existing challenges of delivering high-quality care.^20^ While half (8/16) of the CIN NBUs (often in large county hospitals) had a paediatrician dedicated to the NBU, the other half had a paediatrician who was also responsible for providing daily services to the paediatric wards. Only 7/16 NBUs had at least one neonatal nurse. Upgrading these facilities means that HRH challenges must be addressed in tandem to efforts to upgrade the equipment.^14 27^

There is high in-patient neonatal fatality in the NBUs studied across all birth weight bands. However, ELBW (<1000 g) with median mortality of 80% is almost five times higher that of HICs, while median mortality of VLBW (1000–1499 g) of 40% is about 10 times higher that of HICs.^28^ Given the limited resources in Kenya and many LMICs, it seems the considerable room for improvement in survival of VLBW infants should be given greater priority than efforts to tackle mortality in those with birth weights <1000 g. However, it is also worth noting that in our study, two-thirds of the babies who died had a birth weight >1500 g, with intrapartum-related complication being the leading cause of death. This is a reversal of what is observed in HICs where babies with GA <33 weeks account for about four-fifths of neonatal deaths.^28^

Majority (90%) of inborn neonates in the NBUs studied are admitted on their first day of life, and three out of every five deaths in the NBUs occur on the first day of admission. Poor quality of intrapartum and immediate postnatal care makes the day of birth the riskiest period for a neonate. WHO has developed guidance for continued care along the life course by integrating maternal and newborn care and promoting maternal perinatal death surveillance and response. However, the ‘P’, other than newborn resuscitation, is a weak component of these strategies.^29–31^ As in other LMICs, but in contrast to HICs, congenital conditions are not among the top 5 causes of admissions/deaths in Kenyan NBUs.^28 32^ This could be explained by the high numbers of preventable causes of deaths (>95%) in this study.

There are several limitations to this study. First, these 16 study hospitals are not a representative sample of Kenyan public hospitals. So, we cannot ascertain generalisability of estimates, although there have been other reports on high NBU mortality in Kenya.^33^ Second, we have used routine data from records reviewed. These can prove to be inaccurate when coupled with missing data and imprecise diagnoses due to limited diagnostic capacity in these low-resource settings. We tried to address these problems by using a stringent data quality assurance system and building leadership in the facilities to improve documentation practices.^6 10 11^ However, we used syndromic diagnoses. For example, diagnosis of neonatal sepsis was based was on clinical signs as described by WHO and national clinical guidelines as all hospitals had limited capacity and half had no capacity to do blood cultures.^34 35^ We used birth weight in our analysis instead of GA. LBW, though accurate, is a composite measure of SGA and prematurity and the proportion that are SGA/preterm will influence disease pattern and outcome. The different survival rates among the VLBWs could, in part, be due to variation in occurrence of SGA with lower mortality in places with a high prevalence of SGA. We did not collect data on stillbirths, but recent work by Hagel *et al* 36 indicates that there may be misclassification of very early deaths as stillbirth in the study setting, which may result in our data underestimating NBU mortality rates. Lastly, we excluded the ‘outborn neonates’ in morbidity and mortality analysis due the variable admission policies for these neonates across hospitals; thus, our data cannot be generalised to the whole population of the NBUs. However, what we have done is to highlight that hospitals must be prepared to provide high-quality care for large numbers of small and sick neonates, but often they are not.^20 22 29 36^

In conclusion, neonatal fatality is unacceptably high, but the varied KMC and NBU admission policies challenge comparison of fatality rates across facilities. The fact that almost all the deaths are preventable offers opportunities to improve newborn survival. Care of the small and sick newborn must be included in efforts to strengthen peripartum care, and this goes well beyond neonatal resuscitation, which is the current ‘signal newborn care function’ for comprehensive emergency obstetric and newborn care training. Nevertheless, in view of the high burden of intrapartum-related complications, more work needs to be done on neonatal resuscitation training of the care providers in these health facilities. Neonates comprise almost half of all admissions and two-thirds of the deaths in the paediatric age group (0–13 years) in the hospitals studied. Thus, there is need for governments to prioritise newborn care services and appropriate resources and staffing as a key element of evidence-based hospital care.^15^ Intentional efforts must therefore be made to support generation and capture of high-quality patient data that are credible, complete, analysable and provide opportunities for learning. CIN has enabled generation and use of local clinical information and aims to promote adoption of better practices and wider health system improvements and performance monitoring as part of efforts to reduce neonatal mortality.^11^

## Data Availability

Data are available on request. Data for this report are under the primary jurisdiction of the Ministry of Health in Kenya. Enquiries about using the data can be made to the KEMRI-Wellcome Trust Research Programme Data Governance Committee.
