# Supplementary material for: Neonatal mortality in Kenyan hospitals: a multisite, retrospective, cohort study
Source: BMJ Glob Health. 2021 May 31;6(5):e004475. doi: 10.1136/bmjgh-2020-004475 (PMC8169483; doi:10.1136/bmjgh-2020-004475)
Supplement: Supplementary data [file bmjgh-2020-004475supp001.pdf]

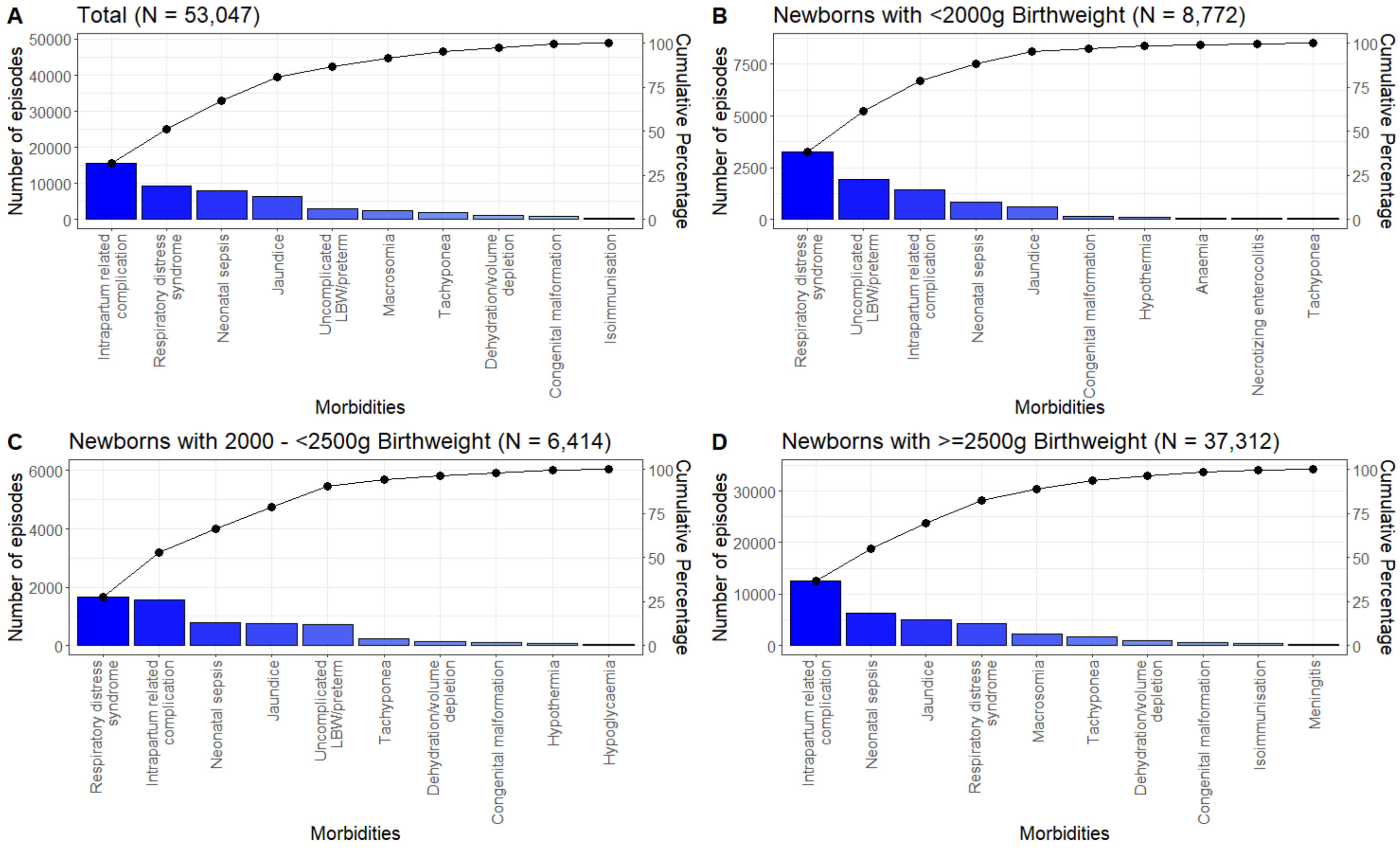

Figure S1 : Prevalence of morbidity episodes among the inborn neonates (Population B) stratified by birthweight in all the 16 NBUs

a. *Clinical diagnosis as recorded by the clinician without necessarily supported by laboratory or radiological investigation*

b. *Disease episodes – each diagnosis is counted as an episode, thus patient comorbidities had more than one disease episode.*

c. *Uncomplicated LBW/prematurity was entered as an admission diagnosis if there was no other comorbidity recorded,*

d. *'Intrapartum related complications 'includes clinical diagnosis of birth asphyxia, hypoxic ischaemic encephalopathy and meconium aspiration*
