# Supplementary material for: Neonatal mortality in Kenyan hospitals: a multisite, retrospective, cohort study
Source: BMJ Glob Health. 2021 May 31;6(5):e004475. doi: 10.1136/bmjgh-2020-004475 (PMC8169483; doi:10.1136/bmjgh-2020-004475)
Supplement: Supplementary data [file bmjgh-2020-004475supp002.pdf]

Table S1: Distribution of morbidity episodes/reason for admission among all the 41,657 inborn neonates (population B) admitted in the NBUs of all the 16 CIN hospitals during the study period.

Diagnoses/Reasons for admission*	**Total**	**Birthweight <2000g**	**Birthweight: 2000 to <2500g**	**Birthweight>=2500g**
Intrapartum related complication	15695	1450	1551	12534
Respiratory distress syndrome	9284	3285	1648	4278
Neonatal sepsis	8038	832	790	6301
Jaundice	6420	589	751	5009
LBW/immature	2863	1954	732	142
Macrosomia	2324	8	10	2294
Tachyponea	1988	51	220	1708
Dehydration/volume depletion	1065	15	126	908
Congenital malformation	883	163	107	600
Isoimmunisation	450	29	41	377
Hypothermia	434	105	78	247
Meningitis	359	20	40	290
Hypoglycaemia	292	46	54	192
Caput succedaneum	287	5	9	273
Admitted for observation	284	2	19	259
Difficulty feeding	261	9	34	217
Birth injury	259	11	18	228
Fever	182	2	8	172
Convulsion	177	1	8	165
Necrotizing enterocolitis	171	56	18	97
Twin delivery	166	10	40	115
Nose/nasal sinuses illnesses	141	3	9	127
Anaemia	129	58	14	57
Pneumonia	119	34	9	73
Choriomniotis	82	5	5	72
Gastrointenstinal illnesses	80	14	23	41
HIV exposed	79	2	9	68
other	535	13	43	468
**Total**	**53047**	**8772**	**6414**	**37312**

** Diagnoses made by clinician but imply clinical syndromes or reason for admission in NBU rather than formal diagnoses based on the ICD-10 system*
