# Supplementary material for: Neonatal mortality in Kenyan hospitals: a multisite, retrospective, cohort study
Source: BMJ Glob Health. 2021 May 31;6(5):e004475. doi: 10.1136/bmjgh-2020-004475 (PMC8169483; doi:10.1136/bmjgh-2020-004475)
Supplement: Supplementary data [file bmjgh-2020-004475supp003.pdf]

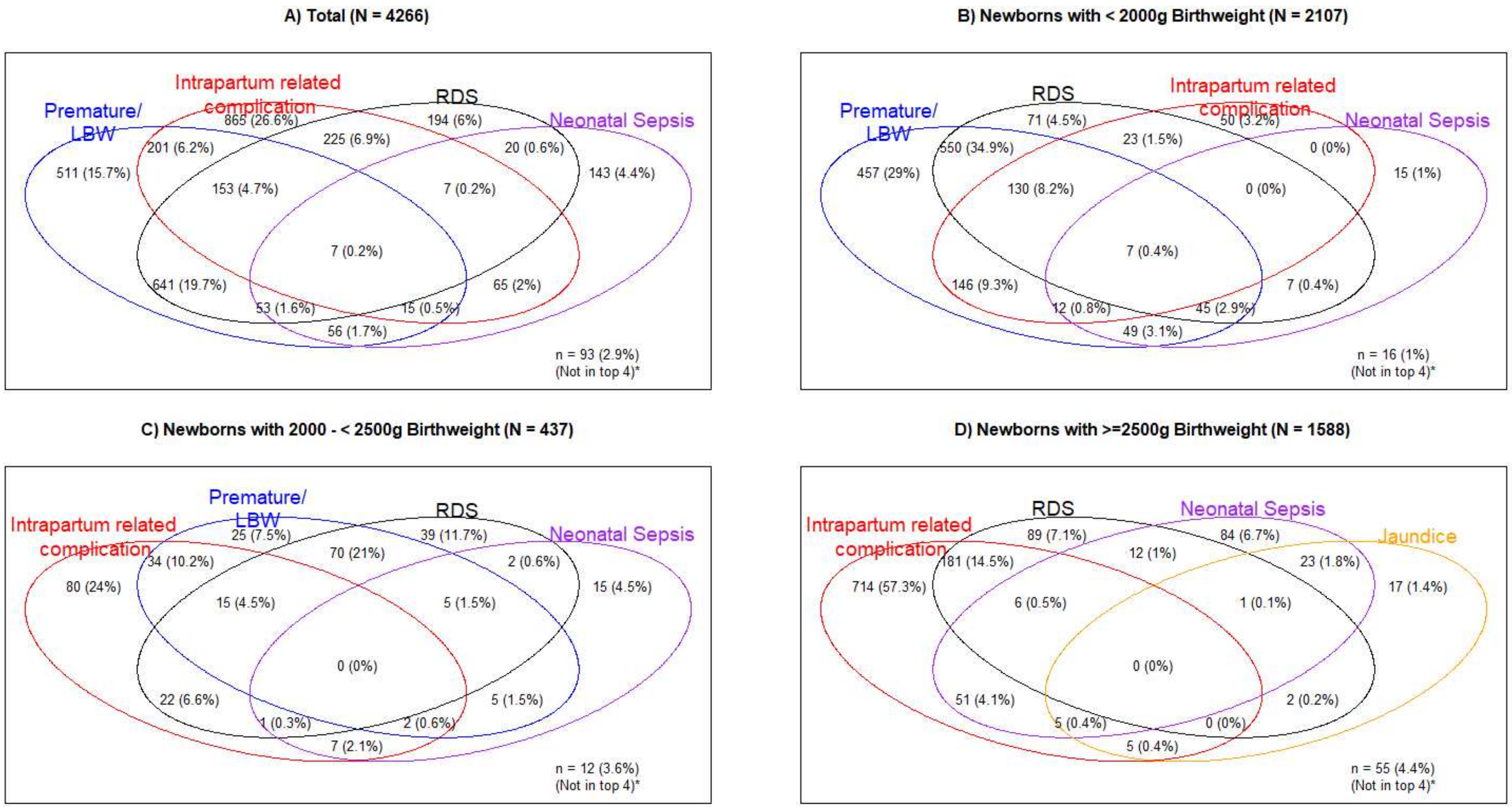

Figure S2: Four leading causes of deaths among the inborn neonates in the 16 NBUs (Population B). **A** - causes of death among all the 4266 inborns who died during the study period, **B, C & D** -causes of deaths among inborn neonates with birthweight <2000grams, 2000to <2500 grams and ≥ 2500grams respectively.
*disease episodes, associated with neonates who died, that were none of the four leading diagnoses as either a primary or comorbid condition.
Abbreviations: LBW- Low birth weight, RDS- Respiratory distress syndrome
