# Supplementary material for: Neonatal mortality in Kenyan hospitals: a multisite, retrospective, cohort study
Source: BMJ Glob Health. 2021 May 31;6(5):e004475. doi: 10.1136/bmjgh-2020-004475 (PMC8169483; doi:10.1136/bmjgh-2020-004475)
Supplement: Supplementary data [file bmjgh-2020-004475supp004.pdf]

Table S2: Mortality prevalence by birthweight category among the inborn neonates in the NBUs of the 16 CIN hospitals (Population B)

	**< 1 Kg**	**1 - < 1.5 Kg**	**1.5 - < 2 Kg**	**2 - < 2.5 Kg**	**2.5 - 4 Kg**	**> 4 Kg**	**Total**
H1	85% (11/13)	58% (25/43)	24% (34/142)	13% (18/138)	12% (16/568)	14% (8/57)	**17% (165/961)**
H2	71% (12/17)	34% (30/87)	17% (21/123)	17% (13/75)	10% (46/468)	8% (3/37)	**15% (125/807)**
H3	95% (39/41)	47% (51/108)	19% (39/204)	12% (36/306)	6% (139/2340)	5% (5/110)	**10% (309/3109)**
H4	81% (25/31)	41% (33/80)	11% (14/132)	6% (7/119)	9% (38/440)	6% (2/35)	**14% (119/837)**
H5	87% (45/52)	50% (93/187)	18% (60/337)	8% (27/323)	12% (151/1275)	3% (9/297)	**16% (385/2471)**
H6	79% (30/38)	40% (31/78)	11% (19/178)	10% (25/249)	5% (67/1312)	3% (3/86)	**9% (175/1941)**
H7	84% (52/62)	60% (100/168)	19% (65/335)	11% (48/442)	6% (132/2329)	2% (5/232)	**11% (402/3568)**
H8	71% (15/21)	38% (19/50)	15% (16/108)	5% (6/131)	6% (35/593)	0% (0/52)	**10% (91/955)**
H9	79% (31/39)	36% (47/130)	22% (51/236)	14% (22/162)	12% (97/777)	6% (3/47)	**18% (251/1391)**
H10	53% (41/77)	32% (58/181)	7% (27/390)	5% (29/551)	3% (106/3616)	3% (7/258)	**5% (286/5073)**
H11	87% (85/98)	49% (143/293)	14% (80/566)	9% (45/512)	7% (164/2240)	1% (5/461)	**13% (522/4170)**
H12	67% (44/66)	32% (55/174)	12% (37/306)	10% (25/241)	10% (95/905)	0% (0/55)	**15% (256/1747)**
H13	80% (28/35)	38% (36/94)	12% (24/206)	5% (15/275)	5% (63/1351)	0% (1/219)	**8% (167/2180)**
H14	86% (6/7)	50% (20/40)	11% (12/109)	13% (11/84)	8% (30/380)	0% (0/21)	**12% (79/641)**
H15	86% (31/36)	44% (45/103)	16% (260/41)	10% (37/389)	4% (73/1849)	1% (2/296)	**8% (229/2933)**
H16	81% (73/90)	42% (125/297)	12% (88/730)	7% (73/1007)	4% (223/5898)	2% (7/360)	**7% (589/8382)**
**Total**	**79% (568/723)**	**43% (911/2113)**	**14% (628/4362)**	**9% (437/5004)**	**6% (1528/26341)**	**2% (60/2623)**	**10% (4132/41166)**
