# Supplementary material for: Neonatal mortality in Kenyan hospitals: a multisite, retrospective, cohort study
Source: BMJ Glob Health. 2021 May 31;6(5):e004475. doi: 10.1136/bmjgh-2020-004475 (PMC8169483; doi:10.1136/bmjgh-2020-004475)
Supplement: Supplementary data [file bmjgh-2020-004475supp005.pdf]

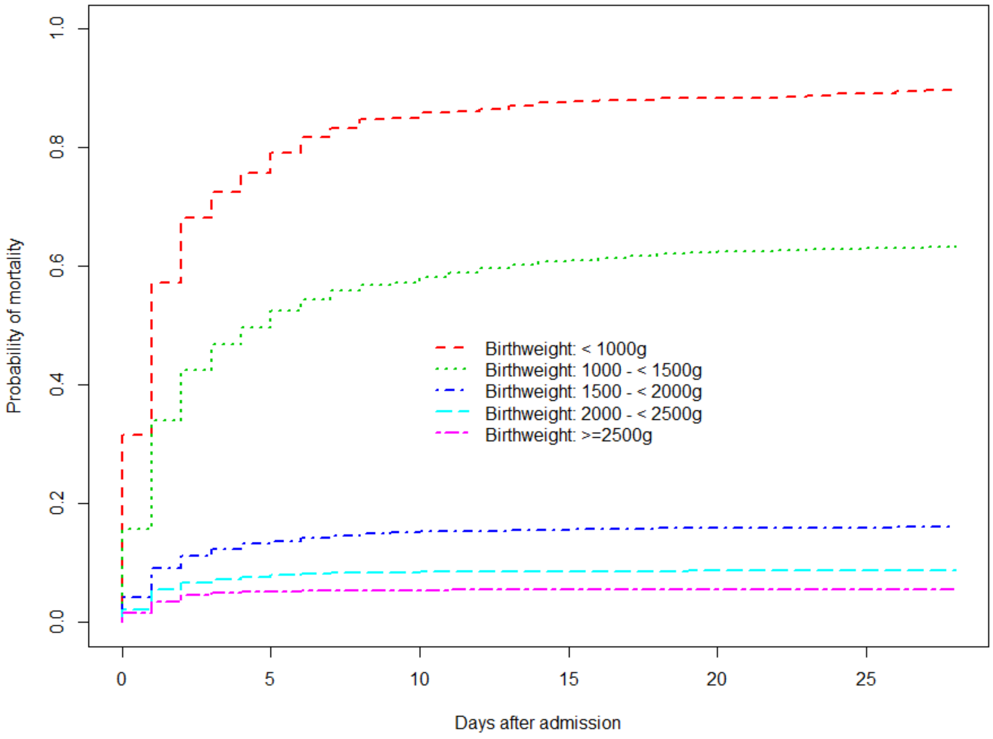

Figure S3: Probability of mortality of the inborn neonates (Population B) by length of stay during 4 weeks of admission in the NBU of 16 hospital in the CIN
